# Comparative study of three types of lymphadenectomy along the left recurrent laryngeal nerve by minimally invasive esophagectomy

**DOI:** 10.1111/1759-7714.13210

**Published:** 2019-12-20

**Authors:** Shuangping Zhang, Peng Zhang, Shiping Guo, Jianhong Lian, Yun Chen, Ailan Chen, Yong Ma, Feng Li

**Affiliations:** ^1^ Department of Cardiothoracic Surgery Tianjin Medical University General Hospital Tianjin China; ^2^ Department of Thoracic Surgery Shanxi Cancer Hospital Taiyuan China; ^3^ Department of Cleaning & Sterilization Shanxi Cancer Hospital Taiyuan China

**Keywords:** Esophageal cancer, lymphadenectomy, minimally invasive esophagectomy, recurrent laryngeal nerve

## Abstract

**Background:**

The objective of this study was to compare three kinds of lymphadenectomy methods along the recurrent laryngeal nerve (RLN) and assess the safety and effectiveness of the new method.

**Methods:**

A total of 194 patients with esophageal cancer who underwent minimally invasive esophagectomy (MIE) at our institution from May 2013 to May 2017 were analyzed retrospectively. According to the method of lymphadenectomy along the left RLN, the patients were divided into three groups: 75 cases underwent the conventional method (A group), 80 cases the skeletonized method (B group) and 39 cases the modified Bascule method (C group). The number of dissected lymph nodes and surgical outcomes were recorded and compared to identify differences among the three groups.

**Results:**

The frequency of metastasis to the LRLN lymph node was 18.6% among all patients, and 12%, 20% and 28% in groups A, B and C, respectively. The number of harvested lymph nodes (total/chest/LRLN/LRLN+) in group B and group C were significantly greater than that of group A, but not significant between group B and group C. The hoarseness rate in group C was 15.4%, which was lower than the rate in group B (21.3%) and higher than the rate in group A (13.3%), but there was no statistical significance.

**Conclusions:**

The new method for lymphadenectomy along the left RLN during MIE in the semi‐prone position is safe and reliable. It provides sufficient lymph node dissection along the left RLN.

## Introduction

More than 50% of esophageal cancer cases worldwide occur in China. Esophageal cancer is the sixth most common cancer in China with 258 000 new cases (6.8% of the total) estimated in 2014, and it is the fourth most common cause of death from cancer with 193 000 deaths (8.4% of the total).^1^

Esophagectomy with radical lymphadenectomy plays the most vital role in treating patients with esophageal cancer. With the development of endoscopic equipment and the progress of surgical technology, minimally invasive esophagectomy (MIE), which is characterized by minimal access, lower postoperative pain, fewer complications and fast recovery, has become the treatment of choice in recent years.^2^

Metastasis of thoracic middle and upper esophageal cancer to superior mediastinal lymph nodes, especially the lymph nodes around the recurrent laryngeal nerve (RLN), is common.^3^ It is essential following lymphadenectomy along the RLN during esophagectomy for accurate pathological staging and prognostic evaluation, but does require more advanced and reliable dissection skills.^4^ Although thoracoscopic lymphadenectomy, particularly along the left RLN, is thought to be a burdensome step, with potential difficulties arising during operative exploration in the left lateral decubitus position, which may also lead to a potential increase in the risk of RLN damage, however, it is considered reliable and feasible in experienced hands.^5^

Currently, either the conventional or skeletonized method are adopted in lymphadenectomy along the RLN. However, these methods are deficient in that the conventional method cannot reflect the real staging due to the small number of lymph nodes. The skeletonized method often leads to aspiration pneumonia and sometimes severe fatal complication due to the high risk of recurrent laryngeal nerve paralysis (RLNP).^6^ Therefore, a new method which can not only satisfy the number of dissected lymph nodes, but also reduce the RLNP, is urgently needed.

Oshikiri *et al*.^7^ introduced the Bascule method for lymphadenectomy along the left RLN during prone esophagectomy for esophageal cancer. The emphasis of this method is that there must be a good understanding of the anatomical concept of esophageal mesenteriolum, the lateral pedicle as a two‐dimensional membrane that includes the left RLN, lymph nodes, and primary esophageal arteries. In order to obtain more lymph nodes along the left RLN, we adopted the modified Bascule method which not only accepted the concept of esophageal mesenteriolum, but lessons were learned from this method and the left RLN was subjected to skeletonization.

In this study, we retrospectively analyzed 194 patients who underwent McKeown MIE, and found that application of a modified Bascule method during lymphadenectomy along the left RLN in MIE is safe and effective for reducing the risk of RLNP.

## Methods

### Patients

A total of 194 patients with thoracic esophageal cancer who underwent McKeown MIE with extended mediastinal lymph nodes dissection at Shanxi Cancer Hospital from May 2013 to May 2017 were reviewed. All patients were assessed to determine operability by routine preoperative examination, including arterial blood gas analysis, electrocardiography, spirometry, endoscopy, biopsy results, abdominal‐thoracic enhanced computed tomography (CT) and endoscopic ultrasound.

According to the method of lymphadenectomy along the left RLN, the patients were divided into three groups: 75 patients underwent the conventional method (A group), the skeletonized method was performed on 80 cases (B group) and the new modified Bascule method was used on 39 cases (C group).

### Thoracic trocar locations

The patient was intubated with a univent bronchial tube and maintained in a left lateral semi‐prone position. A 12 mm trocar was placed at the seventh intercostal space (ICS) for insertion of a 30 degree angled 10 mm thoracoscope, after carbon dioxide insufflation at 6–9 mmHg. Other trocars were situated with inspection of the pleural space as follows: A 5 mm port was located at the fourth or third ICS along the mid‐axillary line, the second 5 mm port was set at the sixth ICS of the subscapular line and a 12 mm port was inserted at the ninth ICS of the subscapular line.

### Surgical technique of left RLN lymph node dissection in C group

**Figure 1:**
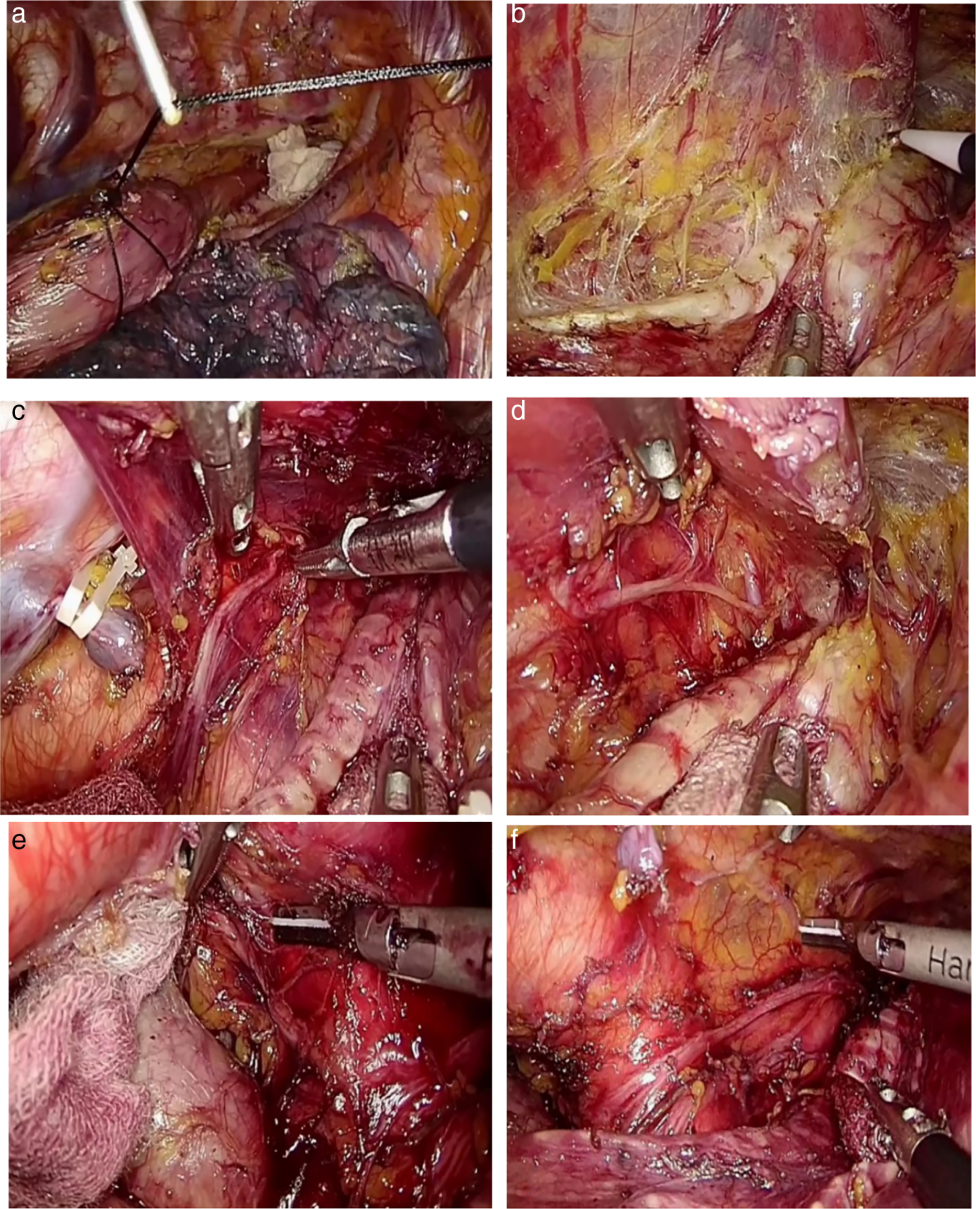
Illustration of lymphadenectomy along the left RLN. (a) A crochet needle is punctured into the thorax to lift the esophagus with double 0# silk suture which has looped the esophagus at the level of the aortic arch. (b) The tracheoesophageal and primary esophageal arteries are identified in the esophageal mesenteriolum. (c) With good countertraction, the left RLN is clearly exposed and separated in the esophageal mesenteriolum above the level of the aortic arch. (d) The paratracheal lymph node is dissected and the left inferior thyroid artery is occasionally visible. (e) When the infra‐aortic arch lymph nodes have been dissected, the initial segment of the left RLN is confirmed. The trunk of the left pulmonary artery under the aortic arch is visible. (f) Following removal of the left RLN lymph nodes, the left RLN is easily clarified as running toward the oral side along the space between the lifted esophagus and trachea. A couple of superior cardiac branches of the sympathetic nerve system were identified. The thoracic duct is preserved.

After mobilizing the middle and lower part of the esophagus, the upper part of the esophagus was partially dissociated from the membranous part of the trachea by cutting the primary and secondary tracheal arteries. In order to reveal the ventral border of the dissection, the tissue was dissected including the left RLN and lymph nodes with the associate adipose tissue along the trachea and the left bronchus (Fig 1a–f). The trachea was then rotated with a grasper holding a small gauze in order to explore the left aspect of the trachea and the left main bronchus. A crochet needle was used to puncture the thorax in order to lift the esophagus using double silk 0‐suture and loop the esophagus at the level of the aortic arch. The puncture point was at the fifth ICS between the vertebral body and right scapula. The thread that had looped the esophagus was then pulled out. The outside of the thorax part of the thread was pulled up by an assistant to lift the esophagus.

With good countertraction, the tissue that included the left RLN and the lymph nodes with their associated fat was also drawn through, the esophageal mesenteriolum (lateral pedicle) was extended, the left RLN was easily identified and clarified the running direction in the space between the lifted esophagus and trachea. The left RLN was then exposed and separated in the esophageal mesenteriolum above the level of the aortic arch. The thoracic duct was preserved. A few esophageal branches of the left RLN were sharply transected. The subaortic lymph nodes around the initial segment of the left RLN were dissected. The recurrent portion of the left RLN, left vagus nerve, one or two left bronchial arteries were identified and preserved on the face of the trunk of the left pulmonary artery between the aortic arch and the left main bronchus. The tissue was retained between the left RLN and vascular sheath of the aortic arch and left subclavicular artery.

### Conventional (A group) and skeletonized (B group) methods

The esophagus was pulled to the ventral side and the trachea simultaneously rotated by a grasper with a small gauze after circumferentially mobilizing the esophagus from the diaphragmatic reflection to the thoracic inlet. The left RLN was frequently found embedded in the neighboring tissue between the trachea and aortic arch. Once the left RLN had been identified, the surgeon maintained hold of the lymph nodes with his left hand, and the lymph nodes along the left RLN were then carefully dissected. In group A, only the dorsal side of the left RLN was dissected. However, in group B, the left RLN was skeletonized and lymph nodes including the neighboring adipose were completely dissected around the left RLN.

### Statistical analysis

Clinical and pathological characteristics were expressed as mean ± standard deviation for continuous variables and described as numbers for discrete variables. Between groups of comparisons for continuous variables were examined by Student's *t*‐test or Mann‐Whitney U tests and discrete variables analyzed using the chi‐square or Fisher's exact test. A *P*‐value less than 0.05 was considered statistically significant. All statistical analyses were performed using SPSS version 16.0.

## Results

### Patient and tumor characteristics

Patient and tumor characteristics are shown in Table 1. There were no significant differences among the three groups in age, gender, tumor location, differentiation, tumor size, lymphatic, venous and nerve invasion.

**Table 1:** Clinical pathological characteristics of the 194 patients with esophageal cancer

	A group	B group	C group	*P*‐value
*N*	75	80	39	
Age (years)	60.67 ± 8.17	61.51 ± 6.88	58.90 ± 7.87	0.934
Gender (male/female)	52/23	53/27	23/16	0.540
Location of the main tumor (upper/middle/lower)	3/54/18	4/59/17	4/29/6	0.312
Differentiation (G1/G2/G3)	13/38/24	13/44/23	4/24/11	0.804
T category				0.911
T1	17	14	9	
T2	18	25	11	
T3	34	34	15	
T4	6	7	4	
N status				0.290
N0	44	40	20	
N1	14	24	11	
N2	9	8	5	
N3	8	8	3	
Lymphatic and venous invasion				0.737
No	61	61	31	
Yes	14	19	8	
Nerve invasion				0.397
No	73	74	37	
Yes	2	6	2	
Tumor size (cm)	3.41 ± 1.45	3.75 ± 1.56	3.55 ± 1.38	0.884
<3	21	20	10	
3–5	48	53	25	
>5	6	7	4	

### Left recurrent laryngeal nerve lymph node metastasis

Among the 194 patients with esophageal cancer, 90 experienced lymph node metastasis (46.4%) (group A [41.3%], group B [50%] and group C [48.7%], respectively). The mean number of harvested total lymph nodes in group A was 16.91 ± 6.47, which was significantly lower than that of group B (24.01 ± 7.15) and group C (24.10 ± 10.01). Similarly, the mean number of thoracic lymph nodes in Group A was 9.09 ± 3.93, which was significantly lower than that of group B (14.02 ± 5.37) and in group C (14.49 ± 5.39) there was no significant difference. The mean number of harvested total lymph nodes or thoracic lymph nodes did not differ between groups B and C.

A total of 609 LRLN lymph nodes were detected, and the mean number of harvested LRLN lymph nodes in group A was 1.59 ± 0.84, which was significantly lower than that of group B (4.02 ± 1.96) or group C (4.31 ± 2.14). Similarly, the mean number of positive LRLN lymph nodes in group A was 0.13 ± 0.38, which was significantly lower than that of group B (0.32 ± 0.74) or Group C (0.46 ± 0.91). The mean number of harvested LRLN lymph nodes or positive LRLN lymph nodes did not differ between group B and C (Table 2).

The frequency of metastasis to the LRLN lymph node was 18.6% among all patients, and 12%, 20% and 28% in group A, B and C, respectively (Table 3).

**Table 2:** Surgical outcomes of the patients using three different methods

	A group	B group	C group	*P*‐value
Operation time (minutes)				
Chest	75.8 ± 30.11	90.5 ± 18.7	85.9 ± 19.2	0.001
Estimated blood loss in the chest	53.7 ± 18.5	60.7 ± 25.4	60.9 ± 19.8	0.180
Number of dissected lymph nodes				
Total	16.91 ± 6.47	24.01 ± 7.15	24.10 ± 10.01	0.001
Chest	9.09 ± 3.93	14.02 ± 5.37	14.49 ± 5.39	0.001
Along the left RLN	1.59 ± 0.84	4.02 ± 1.96	4.31 ± 2.14	0.001
Positive number of lymph nodes				
Total	1.71 ± 3.04	2.08 ± 3.85	1.95 ± 3.92	0.812
Chest	0.95 ± 2.11	1.32 ± 2.68	1.31 ± 2.60	0.590
Along the left RLN	0.13 ± 0.38	0.32 ± 0.74	0.46 ± 0.91	0.035
Mortality	0	0	0	
Postoperative morbidity related to the left RLN				
Hoarseness	10	17	6	0.265
Pneumonia	6	8	3	0.912
Anastomotic leakage	11	16	5	0.523
Chylothorax	1	0	0	0.460

**Table 3:** Lymph node metastasis status in the three groups

	Lymph node metastasis rate		Degree of lymph node metastasis	
Group	Total	LRLN	Total	LRLN
A	41.3% (31/75)	12% (9/75)	10.1% (128/1268)	8.4% (10/119)
B	50% (40/80)	20% (16/80)	8.6% (166/1921)	8.1% (26/322)
C	48.7% (19/39)	28% (11/39)	8.1% (76/940)	10.7% (18/168)

### Postoperative complications

The hoarseness rate in group C was 15.4% (6/39), which was significantly lower than that in group B (21.3%, 17/80) (x^2^ = 5.594, *P* = 0.018), but similar with that in group A (13.3%, 10/75) The median intrathoracic operation time in both group B (90.5 ± 18.7*, P* = 0.001) and group C (85.9 ± 19.2, *P* = 0.001) were significantly longer than group A (and 75.8 ± 30.11). However, there were no significant differences between group B and group C (*P* = 0.651).

There was no significant difference among the three groups in blood loss, hospital length of stay, and complication profile.

## Discussion

Cuschieri *et al*. first reported thoracoscopic esophagectomy for esophageal carcinoma in 1992.^8^ This approach was very attractive because it offered minimal invasion, high‐definition magnified view and superb visualization enabling the operation to be more accurate and meticulous.^9^, ^10^

Superior mediastinal lymph nodes in esophageal cancer are thought to be highly involved, especially along the bilateral RLN,^11^ and complete dissection recommended in order to improve survival rate and decrease local recurrence.^12^ Ninomiya *et al*. 13 proved that it was beneficial for patients with lymph node metastasis around bilateral RLNs by reviewing their 10‐year experience. Niwa *et al*. 3 reported that the frequency of metastasis to the RLN lymph node was 15.8% among 342 patients and 40.5%, 16.2% and 6.6% in patients with upper, middle and lower ESCC, respectively. Tan *et al*.^11^ demonstrated that the incidence of RLN lymph node involvement was 26% (66/254), including the right side 20.9% (53/254), left side 8.7% (22/254) and bilaterally 3.5%(9/254). Oshikiri *et al*.^14^ described that the incidence of lymph node metastasis along the LRLN was 22%. In our study, the incidence of LRLN lymph node involvement was 18.6% (12%, 20% and 28% in group A, B and C, respectively), which is similar to the descriptions of previous studies.

Currently, dissection of LNs along the RLN is seen as a technically demanding and time‐consuming procedure with no room for error which may increase the risk of RLN damage, particularly on the left side.^15^ Koyanagi *et al*.^6^ found that the left RLNP accounted for 82.7% of 198 unilateral RLNP. Sato *et al* 16 also showed the left RLNP accounted for 58.4% of 178 patients with RLNP.

The left RLN originates from the vagus nerve, rounds the aortic arch to running in the tracheoesophageal groove, and is located in a deep and narrow space which is a longer path compared to the right RLN.^17^ The incidence of RLNP after MIE has been shown to range from 0% to 41.9%.^18^, ^19^ The possible cause of this phenomenon is not only related to the extent of lymph node dissection, but also bound up with the methods of postoperative laryngeal examination. To date, there is no standard method for the evaluation of RLNP after MIE both at home and abroad. In fact, the incidence of vocal palsy has been reported to be higher than that of hoarseness when the motility of the vocal cord was directly observed using a laryngoscope.^16^

In our research, the previous cases were not all examined laryngoscopically because some patients had no clinical signs of the larynx. For ease of comparative studies, hoarseness was considered as a primary criterion for evaluating the RLNP. The prevalence of hoarseness in group C was 15.4% (6/39), which was lower than that in group B (21.3%, 17/80) and higher than that in group A (13.3%, 10/75), but the difference was not statistically significant. However, the downward trend in the prevalence of hoarseness in the group undergoing the new method is likely to continue as the number of patients accumulates.

Recent studies^2^, ^20^, ^21^, ^22^, ^23^ from Japan have shown the benefits of MIE in the prone position in terms of better surgical exposure, a larger number of dissected lymph nodes and less blood loss compared to MIE in the left lateral decubitus position. However, most cases undergo MIE in the left lateral decubitus or semi‐prone position in China.^4^, ^5^, ^24^, ^25^, ^26^ It is difficult to obtain better operative exposure around the left RLN in this position because the collapsed right lung and mediastinal organs and structures move to the left side, which deepens the location of the left RLN.

There are several reasons why the left lateral decubitus or semi‐prone position is widely applied for MIE in China. Firstly, the surgical field and anatomical relationship of the posterior mediastinum in this position are very familiar to most thoracic surgeons because it is similar to right transthoracic open esophagectomy. Secondly, this position is still considered safe and necessary during operative procedures. When an emergency occurs, such as a sudden massive bleeding or tracheal injury, an urgent conversion to right thoracotomy can be rapidly performed.^27^, ^28^ Thirdly, anesthetists are skilled at managing these changes in intraoperative respiration and hemodynamics in this position during MIE, and it is also more convenient for airway management.^27^

In this report, the mean number of harvested lymph nodes along the left RLN in the conventional group was 1.59 ± 0.84. The incidence of lymph node metastasis along the left RLN was 12%, which is not enough to affect clinical pathological staging and prognosis. By adopting the skeletonized method, the number of harvested lymph nodes along the left RLN was 4.02 ± 1.96, the incidence of lymph node metastasis along the left RLN was 20%, but the prevalence of hoarseness rose to 21.3%, which is higher than that of the conventional group.

It is an important challenge for a surgeon to be able to strike the right balance in performing a lymphadenectomy along the left RLN during MIE without increasing the risk of postoperative complications. The tissues between the lateral side of the left RLN and vascular sheaf of the left subclavicular artery and the aortic arch were retained in 39 cases. The number of harvested lymph nodes along the left RLN was 4.31 ± 2.14, the incidence of lymph node metastasis along the left RLN was 28%, and the prevalence of hoarseness had decreased to 15.4% compared with group B.

This new method has definite advantages over other procedures. First, by lifting the proximal portion of the esophagus and rotating the trachea, the countertraction was so good that the surgical field of the left RLN was clearly exposed and enabled the surgeon to meticulously dissect, easily identify and isolate the left RLN. Second, Nakajima *et al*. 29 found that no network of lymphatic vessels running craniocaudally and straddling the left RLN were seen through the operating microscope and histological examination using serial sections. Thus, the number of harvested lymph nodes along the left RLN was not reduced via the new technique. Third, the reason that left RLN injuries can be reduced by adopting the modified method may be mainly related to avoidance of excessive dissociation of the left RLN, reducing the influence on the blood supply of the left RLN, and avoiding excessive traction to the nerve.

The new method for lymphadenectomy along the left RLN during MIE in the semi‐prone position is safe and reliable. It provides sufficient lymph node dissection along the left RLN and the rates of RLN palsy could potentially be lower as the number of cases accumulates.

## Disclosure

The authors report that there are no conflict of interests.
